# An open-source semi-automated robotics pipeline for embryo immunohistochemistry

**DOI:** 10.1038/s41598-021-89676-5

**Published:** 2021-05-13

**Authors:** Timothy Fuqua, Jeff Jordan, Aliaksandr Halavatyi, Christian Tischer, Kerstin Richter, Justin Crocker

**Affiliations:** 1 European Molecular Biology Laboratory, Heidelberg, Germany; 2 Collaboration for Joint PhD Degree Between EMBL and Heidelberg University, Faculty of Biosciences, Heidelberg, Germany; 3 Janelia Research Campus, 19700 Helix Dr, Ashburn, VA 20147 USA

**Keywords:** Biological techniques, Biotechnology, Developmental biology

## Abstract

A significant challenge for developmental systems biology is balancing throughput with controlled conditions that minimize experimental artifacts. Large-scale developmental screens such as unbiased mutagenesis surveys have been limited in their applicability to embryonic systems, as the technologies for quantifying precise expression patterns in whole animals has not kept pace with other sequencing-based technologies. Here, we outline an open-source semi-automated pipeline to chemically fixate, stain, and 3D-image *Drosophila* embryos. Central to this pipeline is a liquid handling robot, *Flyspresso*, which automates the steps of classical embryo fixation and staining. We provide the schematics and an overview of the technology for an engineer or someone equivalently trained to reproduce and further improve upon *Flyspresso,* and highlight the *Drosophila* embryo fixation and colorimetric or antibody staining protocols. Additionally, we provide a detailed overview and stepwise protocol for our adaptive-feedback pipeline for automated embryo imaging on confocal microscopes. We demonstrate the efficiency of this pipeline compared to classical techniques, and how it can be repurposed or scaled to other protocols and biological systems. We hope our pipeline will serve as a platform for future research, allowing a broader community of users to build, execute, and share similar experiments.

## Quote

“Rock, robot rock

Rock, robot rock

Rock, robot rock”

– Daft Punk, 2005

## Introduction

Changes in gene expression drive animal development and evolution^1^. Even small changes in expression levels define tissue‐ and cell‐type‐specific functions. Therefore, quantifying small changes in gene expression is critical to understanding developmental biology^2,3^. In the laboratory, experiments analyzing changes in gene expression have been done classically through several approaches, including RNA in-situ hybridization^4,5^, reporter gene assays^6^, or antibody staining^7^. These approaches have been integral to our understanding of gene-regulation, development, and evolution.

Whole-mount techniques are accurate and contain spatial–temporal information, but are limited by their throughput. Conversely, high-throughput techniques such as single-cell sequencing have limited to no spatial information. In order to achieve high-throughput results comparable to and/or approaching genomic techniques, whole-mount technique automation needs to improve.

Automated liquid-handling robots can precisely and accurately screen multiple whole-mount samples in parallel^8,9^. Although these systems exist for embryo staining, they have several shortcomings, including manual embryo fixations, are prohibitively expensive for many groups, and are not scalable or adaptable. Together, this has hampered and limited the quantitative detection of subtle phenotypic changes at scale, limiting our understanding of genotype-to-phenotype relationships.

To address this, we developed *Flyspresso,* a customizable syringe-based microplate washer to carry out *Drosophila* embryo experiments at a higher throughput, and an adaptive feedback confocal microscope pipeline that allows the automated acquisition of samples^10^. Our pipeline automates several critical steps: (1) embryo fixation, (2) vitelline membrane removal, (3) antibody or chemical staining of embryos, and (4) the automated imaging of embryos. The goal of this approach was to streamline classical *Drosophila* embryo fixation, immunohistochemistry, and imaging protocols on 24 embryonic samples per experiment—embracing a “Do It Yourself” (DIY) ethos that could easily be adapted by others^9,11^.

With this pipeline, we tested a library of developmental enhancer variants driving the *lacZ* gene under the control of a minimalized core promoter. The *lacZ* gene product is β-galactosidase, which, when treated with x-gal (5-bromo-4-chloro-3-indolyl-β-D-galactosidase), forms a blue precipitate reporting where the enhancer is active. Second, we used *Flyspresso* to carry out *Drosophila* embryo fixations, which involves bleaching the embryos, fixing them in paraformaldehyde, and removing the vitelline membrane. Third, we programmed *Flyspresso* to wash chemicals in and out of the embryos for β-gal antibody staining^10^.

Here, we outline design details for building similar robotic staining systems, compare the automated protocol to traditional methods, and provide step-by-step instructions on how to operate the imaging plugin. In the future, our design could be customized for specific users with the long-term goals of scientific reproducibility and scalability. We hope that *Flyspresso* will serve as a DIY platform for users to build, operate, and share protocols which extend beyond just fixations and antibody stainings, including in-situ hybridizations and extended towards growing tissue cultures and organoids.

## Results

### Pipeline overview

**Figure 1:**
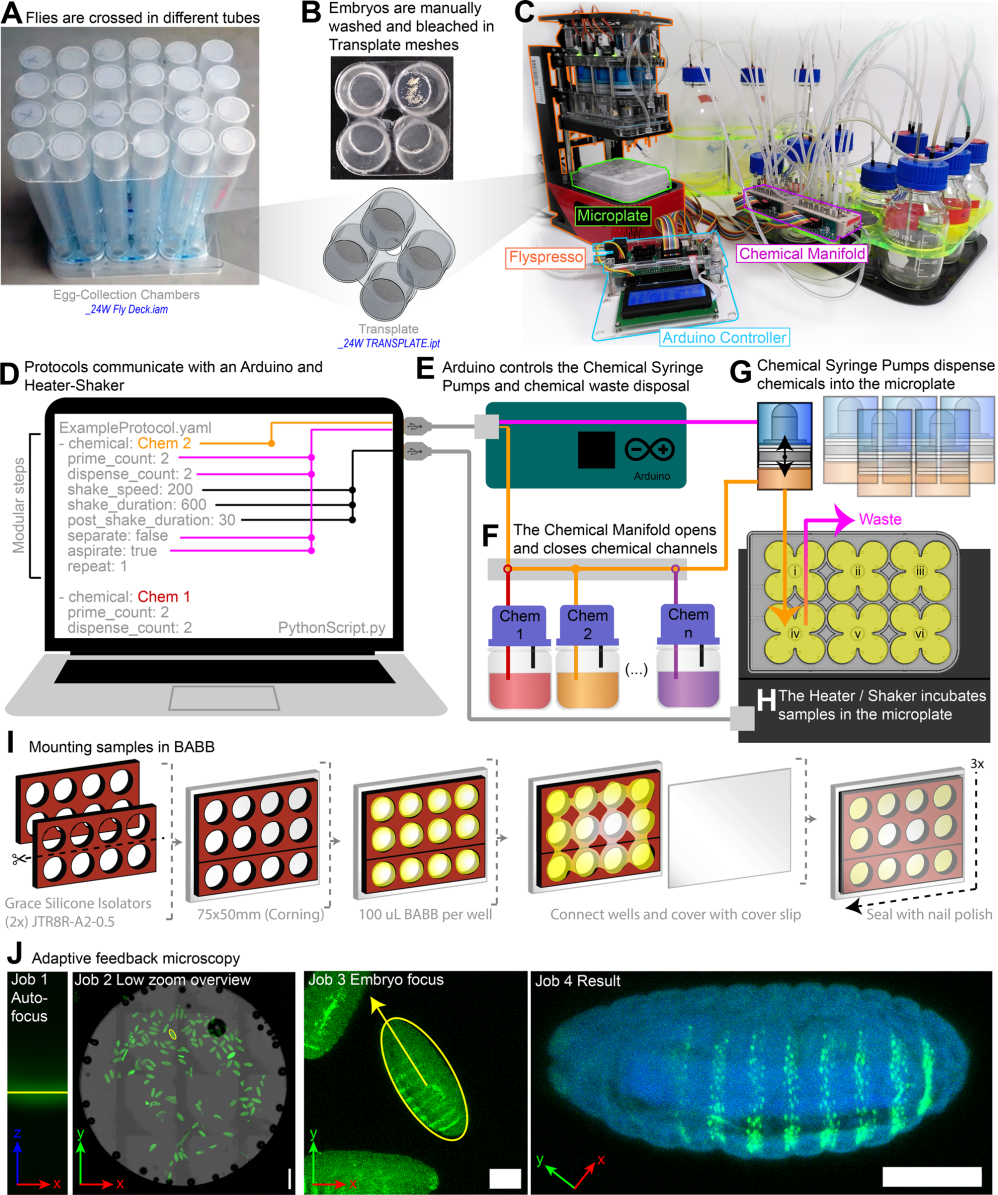
Protocol overview. (**A**) The fly deck (*_24W Fly Deck.iam*) is composed of egg-collection chambers that hold up to 24 different fly samples. (**B**) Four chambers hold a Transplate (*24W TRANSPLATE.ipt*)—detachable for egg collections. Tubes rest on a tray of apple juice-agar media. (**C**) Transplates are loaded into a custom Microplate (green highlight). Microplates are loaded into *Flyspresso* (orange highlight, *_24W.iam*). Chemical inputs are selected with the Chemical Manifold (*24W CHEMICAL MANIFOLD ASSAY.iam,* magenta outline), and an Arduino microcontroller and computer (blue highlight) control the protocols. (**D**) *Flyspresso* protocols are formatted as yaml files that are interpreted by a python script. The protocols can be easily called or written in modular steps. Within each step, which chemical is specified, if the chemical should be primed to clear the tubing, how many times the chemical will be dispensed to the microplate, how fast and long the samples will incubate in the chemical, a wait time after shaking, and whether chemicals are separated (removed from the upper surface) or aspirated (removed from the base of the well), and if the step should be repeated or not. (**E**) The computer sends the protocol to an Arduino microcontroller, which controls both the *Flyspresso* Chemical Syringe Pumps and the Chemical Manifold. (**F**) The Chemical Manifold opens and closes chemical channels based on the protocol step. The open chemical channel is pulled into *Flyspresso* by the Chemical Syringe Pumps. (**G**) The Chemical Syringe Pumps pull chemicals from the Chemical Manifold and dispense them into the microplate below. (**H**) The microplate rests on top of the Heater/Shaker device, which incubates samples at different speeds and temperatures based on the protocol controlled by the computer. (**I**) To mount the embryos, divide a Silicone Isolator and apply it with an additional Isolator to a microscope slide. Add 100 μL of embryos and BABB (yellow) to each well. **c** Allow embryos to sink. Connect the wells with BABB and cover with a cover slip. Seal the perimeter with three coats of clear nail polish. (**J**) The automated adaptive feedback pipeline performs sequentially four imaging “jobs”: Autofocus, Low zoom overview, Embryo focus, and Result. The first three image types are automatically processed to trigger the following jobs at the right locations. “Autofocus” identifies the reflection of the coverslip (green), “Low zoom overview” does a tile scan across each well and identifies samples (green). “Embryo focus” performs a fast 3D embryo scan through the embryo to refine lateral (xy) positions, identify the axial (z) position and the angle of rotation for the embryo. “Result” is a high-resolution confocal scan through the embryo, generating the final image. Scale bars = 100 µm.

We describe here, our pipeline for *Drosophila* embryo collection and fixation, automated x-gal or antibody staining, clearing and mounting the embryos in Benzyl alcohol benzyl benzoate (BABB) for deep-tissue imaging^12^, and automated confocal imaging used in our previous work^10^. An overview of the pipeline is illustrated in Fig. 1.

### Egg collection and chorion membrane removal

We developed an apparatus to hold up to 24 different fly strains in separate plastic tubes (Fig. 1A). The tubes are capped on both sides with a mesh called a Transplate that can be attached or detached to easily transfer flies gassed in CO_2_ into the tubes. The tubes, now capped on both sides with Transplates and filled with flies, rest on an apple juice-agar with a small amount of yeast paste. The flies deposit eggs on the mesh of the Transplate resting on the yeast and agar. We found that there is a better egg yield if the flies have acclimated to the chambers for at least 24 h. Because the tubes have Transplates on both ends, the tubes are flipped and placed over a CO_2_ pad, putting the flies to sleep without getting stuck in the yeast paste. Now the Transplates with freshly laid eggs can be easily removed from the tubes.

Standard, commercial chambers can also be used for egg collections. To transfer the embryos manually, use a paintbrush and fly saline to lightly separate the eggs from apple juice-agar and carefully pour the embryos into the Transplates.

Within the Transplates, the embryos are manually washed in a fly saline solution. The Transplates are manually bleached in a petri dish and afterwards rinsed in water to remove any excess bleach (Fig. 1B). To begin chemical fixations for antibody staining or to carry out x-gal staining, the Transplates are loaded into the custom microplates.

### Chemical fixations, x-gal staining, and antibody staining

The microplates are loaded into *Flyspresso* for automated fixation (Fig. 1C). The fixation protocols are different for x-gal and antibody staining. For x-gal staining, the fixative solution is formaldehyde-based. After fixation, the samples are washed in PBT, and treated in an x-gal staining solution. Once developed, embryos are removed, washed in PBT, and imaged either manually or using the imaging pipeline.

For antibody staining, the fixative solution is paraformaldehyde-based. After fixation, embryos are treated with methanol to undergo isotonic shocking, which removes the vitelline membrane, and the embryos are repeatedly washed in methanol. Embryos are then rehydrated in PBT, blocked in a blocking solution, stained with primary antibodies, blocked again, stained with secondary antibodies, and serially dehydrated into ethanol. See “Methods” section for fixation and staining protocol details.

Protocols for the *Flyspresso* robot are operated by an attached computer (Fig. 1D). With a python interpreter, easy-to-write and read YAML-based protocols communicate with an Arduino (Mega 2560) microcontroller and a separate heater/shaker device (Q-Instruments, 3000 T-ELM). The protocols are written step-wise, where each step specifies the following: which chemical is used, whether the chemical is primed to clear the tubing, how often a fixed volume of the chemical is pushed into the microplate, the speed and duration the samples shake and incubate in the chemical, the post-shake duration, if the chemical is removed from the upper phase (separate) or from the base (aspirate), and how many times the step repeats.

Information from each step regarding the syringes and desired chemicals is sent to the Arduino (Fig. 1E). The Arduino communicates with the Chemical Manifold to select which chemical can be drawn into *Flyspresso* (Fig. 1F), and controls the movement of the Chemical Syringe Pumps in *Flyspresso* (Fig. 1G). The Chemical Syringe Pumps draw the selected chemical into the system and pushes it into the microplate filled with embryos. The computer communicates with the heater/shaker device regarding the specific temperatures, shake speeds, and how long the samples incubate (Fig. 1H). For the fixation and staining protocols demonstrated here, we did not program temperature conditions.

### BABB clearing and mounting antibody-stained samples

Benzyl alcohol benzyl benzoate (BABB) is a clearing media used for deep-tissue imaging^12^. We manually clear and mount the *Drosophila* embryos in BABB (Fig. 1I) using Silicone Isolators (Grace) to separate the wells, cover the slide with a cover slip, and seal the edges with nail polish. (See methods for further details).

### Automated adaptive feedback 3D confocal microscopy

To complement the ability to process embryos with this increased capacity, we developed a confocal imaging pipeline (Fig. 1J). The pipeline is composed of four different sets of stepwise imaging parameters, or “Jobs”. Job 1 (Autofocus) performs an x–z scan in reflection mode to identify the axial position of the coverslip (green horizontal bar). The microscope navigates the stage so samples are in focus for the next job. Job 2 (Low zoom overview) takes a tile scan (grid) of images and stitches them together to get an overview of the embryos (green) within each entire well. Embryos are either automatically or manually identified. Job 3 (Embryo focus) navigates the stage to each selected embryo and scans quickly through the sample to identify the center in 3D and the orientation of the embryo (yellow ROI surrounding embryo). Job 4 (Result)—the final job—takes this information to acquire a high-resolution stack completely through the embryo.

We opted to use a point scanning confocal microscope, as they are available in most core microscopy facilities and provide the flexibility to adjust the size of the field of view and resolution modalities depending on the demand. To automate entire image acquisition routines, the adaptive feedback microscopy pipeline was implemented on a Zeiss LSM 880 confocal microscope. The extension of the previously developed Java library (https://git.embl.de/halavaty/AutoMicTools) has been programmed for controlling the workflow, running image analysis on automatically acquired images and triggering required imaging modalities on the microscope via the MyPic macro^13^, which was used as a communication interface to the microscope software. The implemented feedback microscopy protocol is available as a Fiji plugin that can be easily adopted to other microscopes that have a programming interface to automatically move a stage and trigger preconfigured acquisition procedures. Furthermore, confocal microscopes are stable, robust, and it is possible to scale the protocol across microscopes.

### The components and manifolds of *Flyspresso*

**Figure 2:**
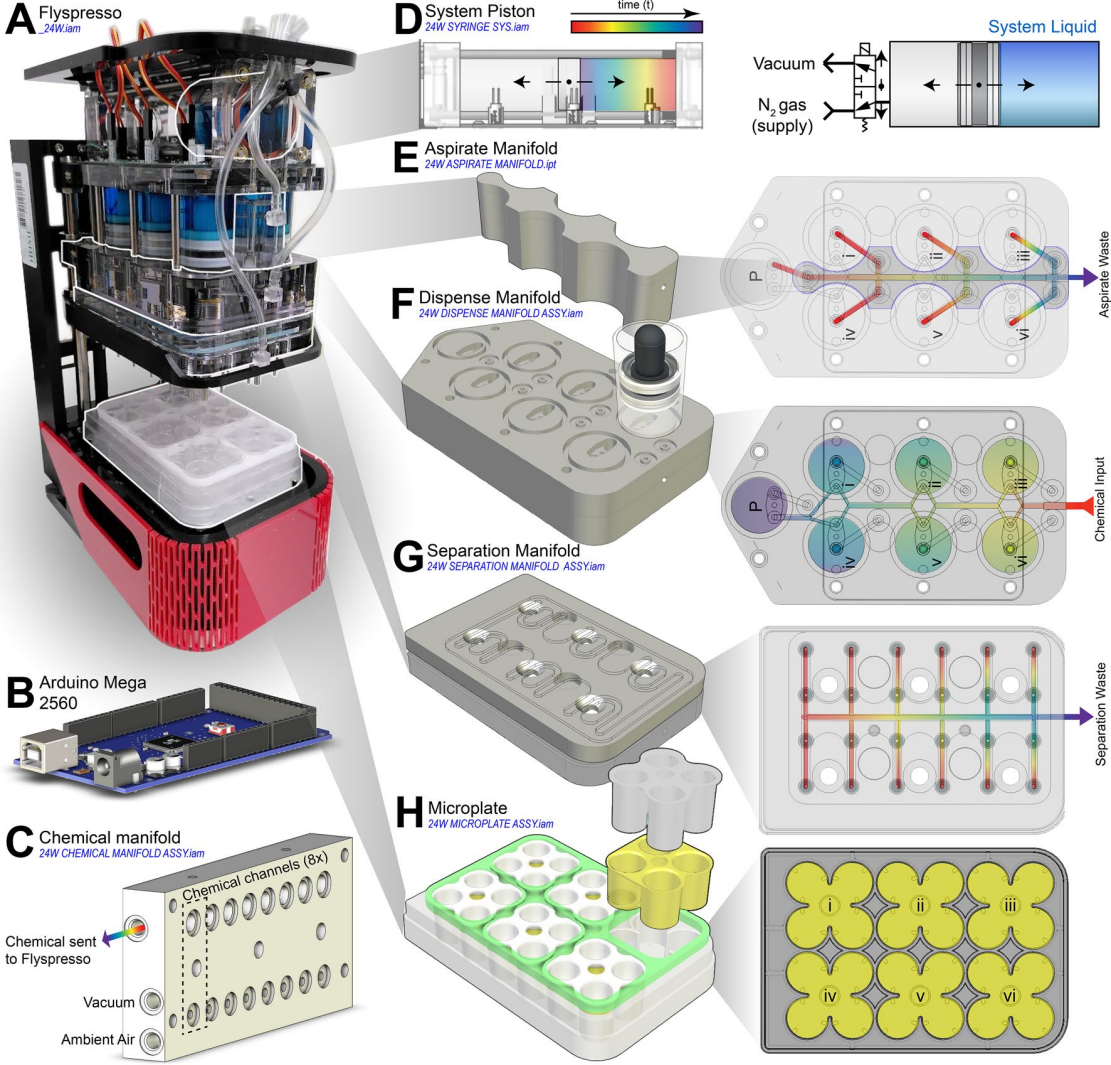
Flyspresso components and design. (**A**) Photograph of the *Flyspresso* robot (_24W.iam), composed of (from top to bottom) the System Piston, Aspirate Manifold, Dispense Manifold, Separation Manifold, Microplate, and the Heating and Shaking Device. (**B**) The syringes and the chemical manifold are controlled by an Arduino Mega 2560 microcontroller, which communicates internally with a python interpreter on a computer with the specific protocol. (**C**) The Chemical Manifold (24 W CHEMICAL MANIFOLD ASSY.iam) can be expanded to the number of channels needed for each experiment. Each chemical channel is controlled by a solenoid valve, which is dictated by the Arduino microcontroller. Chemicals not in use are held via vacuum, and the desired chemical is pulled into *Flyspresso* when the solenoid valve switches to ambient air (see also Fig. 3). (**D–H**) Exploded-view of *Flyspresso*. (**D**) Schematics of the System Piston. Color schematic from red to violet corresponds with the piston’s position when pulling liquid into the system (24 W SYRINGE SYS.iam). The System Piston moves a System Liquid to control the other syringes. Movement is controlled by a gas and vacuum supply (see also Fig. 3). (**E**) (Left) The Aspirate Manifold (24 W ASPIRATE MANIFOLD.ipt) sits between the Chemical Syringe Pumps. (Right) Top view of the Aspirate Manifold laying on the Dispense Manifold. Chemical waste is pulled through the Aspirate Manifold to a waste container. (**F**) (Left) The Dispense Manifold (24 W DISPENSE MANIFOLD ASSY.iam) holds a Priming Syringe Pump (P) and 6 Chemical Syringe Pumps (i–vi). (Right) Top view of the Dispense Manifold shows the chemical pathway to either the Priming Syringe Pump (P) or the 6 Chemical Syringe Pumps (i–vi). (**G**) (Left) The Separation Manifold (24W SEPARATOR ASSY.iam). (Right) Top view of the Separation Manifold. The Separation Manifold is equipped with 24 separator tips, which aspirate liquid from the upper interface to Separation Waste. (**H**) The custom microplate (24 W MICROPLATE ASSY.iam) sits below the Separation Manifold and holds 6 Transplates (yellow) (24W TRANSPLATE.ipt) for a total of 24 samples. Seplate Attachments (gray) (24W SEPLATE.ipt) sit above the Transplates and enclose the embryos, separating the upper and lower liquid interfaces.

**Figure 3:**
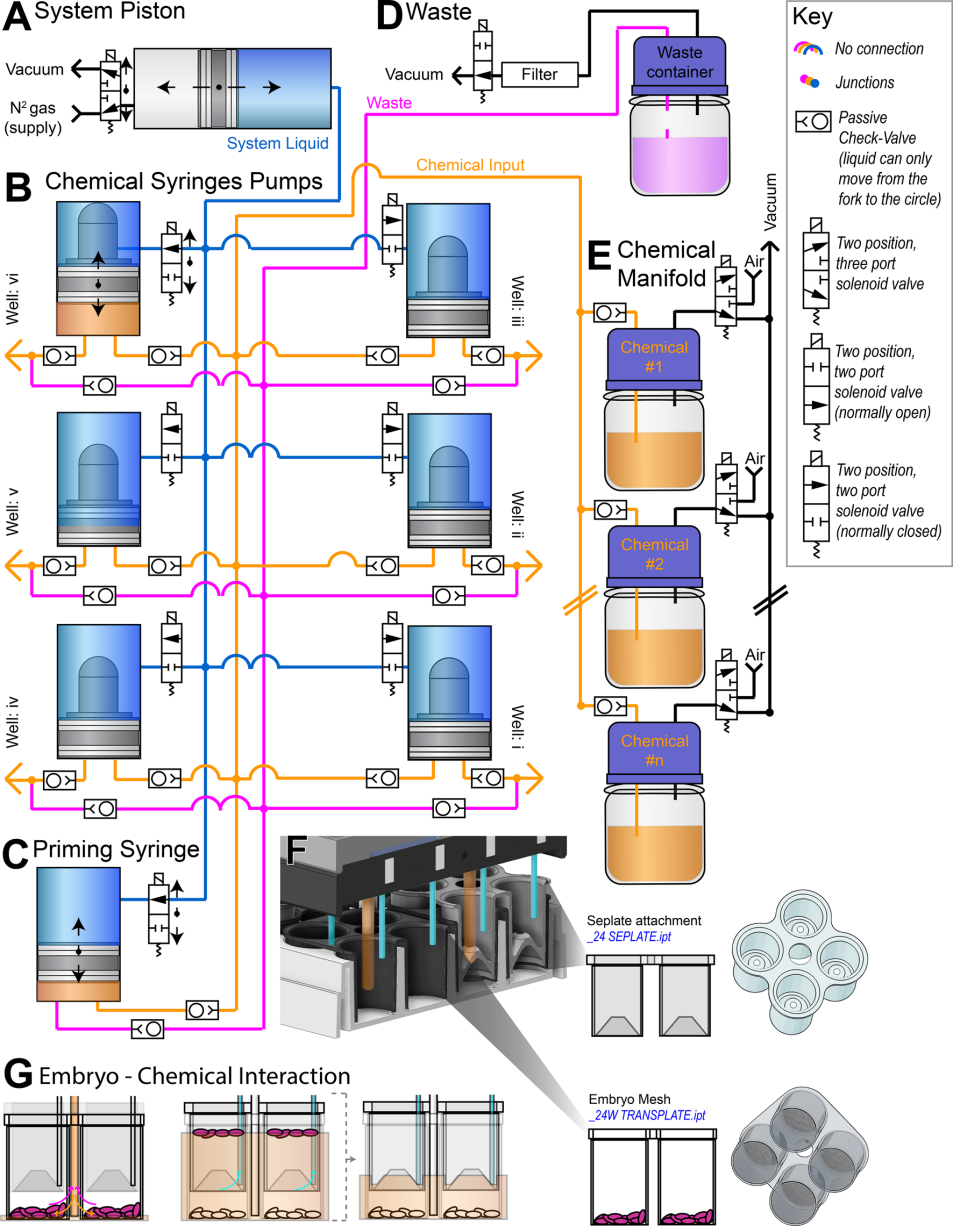
Chemical pathways for *Flyspresso.* (**A**) The System Piston pushes and pulls the System Liquid (blue) to either the Chemical Syringe Pumps or the Priming Syringe Pump. A solenoid valve controlled by the Arduino board, automatically switches between the vacuum and gas supply to move the piston. (**B**) The *Flyspresso* robot is equipped with six Chemical Syringe Pumps. A controllable solenoid valve opens and closes to allow the system liquid to move each Chemical Syringe Pump. The chemical input (orange) is pulled into the syringe and pushed into each corresponding well. Passive check-valves (small rectangles with circles inside) prevent chemicals from flowing in the opposite direction. Chemical waste is also aspirated from the wells (magenta), but does not flow back through the Chemical Syringe Pump. (**C**) The Priming Syringe Pump clears the tubing when a new chemical is used. Similar to the Chemical Syringe Pumps, the Priming Syringe Pump is controlled by a solenoid valve and displacement of the System Liquid. (**D**) Chemical waste from aspiration and priming is carried to waste containers. The waste containers are connected to a solenoid valve, which moves to open or close the vacuum. An activated charcoal filter prevents volatile fumes from being pulled into the vacuum. The entire system is enclosed in a chemical fume hood. (**E**) The Chemical Manifold is a modular and expandable set of solenoid valves attached to reagent bottles. The solenoid valves alternate between ambient air and the vacuum. When not in use, the vacuum prevents the chemical from being pulled into the syringes. When in use, the solenoid valve switches to ambient air, allowing the chemical to pass through a one-way passive check-valve. This valve prevents the chemicals from flowing into and contaminating other containers. (**F**) Cut-through sectioning of the microplate showing the syringe tips for the Chemical Syringe Pumps (orange) and separation tips (blue) from the Separation Manifold (see also Fig. 2). The Transplate (24W TRANSPLATE.ipt) rests on the base of the microplate. Embryos are depicted in magenta and rest in the individual meshes of the Transplates. The Seplate attachment (24 SEPLATE.ipt) inserts above the Transplate and contains inverted cones with small openings for removing chemicals with the Separation Manifold tips. (**G**) (Left) Chemicals from the Chemical Syringe Pumps are loaded into the microplate wells. The tips from the syringes can also aspirate chemicals to chemical waste (magenta). (Middle) During isotonic shocking, embryos that fail to separate and vitelline membranes float to the surface through the Seplate Attachments. The Seplate Attachments nest in the Transplates and contain an inverted cone with a small, one-way orifice. Shocking forces floating membranes and debris through the small, one-way orifice, while successfully shocked embryos do not float and are protected from being aspirated by the Seplate Attachments. (Right) The tips from the Separation Manifold (blue) remove chemicals from this upper surface without disturbing successfully separated embryos at the base of the Transplate (white).

*Flyspresso* is composed of several different components and manifolds (Fig. 2A, see also Fig. 1C). Syringe movement and chemical selection from the Chemical Manifold is controlled by an Arduino Mega 2560 microcontroller (Fig. 2B). The microcontroller is central to the robotics design, providing a small, low-cost interface to a user’s computer or a cloud server. The Arduino microcontroller communicates with electronically controlled solenoid valves on *Flyspresso* and the Chemical Manifold, which open and close to allow syringes to add and remove chemicals to and from the samples. The Arduino also communicates with the Chemical Manifold (Fig. 2C) to select which reagent is transferred to the samples. Chemicals attached to the Chemical Manifold are also controlled by solenoid valves, which can switch between a closed vacuum or open by switching to ambient air (see also Fig. 3).

*Flyspresso* uses a positive-displacement System Piston to displace an internal, ethanol-based System Liquid (Fig. 2D). The System Liquid in turn, is displaced to either the six Chemical Syringe Pumps or the Priming Syringe Pump in liquid communication with the Dispense Manifold (Fig. 2E–F). When *Flyspresso* switches chemicals during a protocol, the Priming Syringe Pump pulls and pushes the new chemical into and out of the system to clear the tubing of residual chemicals. Otherwise, the six Chemical Syringe Pumps pull chemicals to a fixed volume and dispenses them into the microplate. Connected to the same dispense circuitry is the Aspirate Manifold (Fig. 2E). When aspirating chemicals (removing chemicals from the lower interface) from the microplate, chemicals are vacuumed back through the pipette tips attached to the Chemical Syringe Pumps, pass through the Aspirate Manifold and are sent to chemical waste.

*Flyspresso* is also equipped with a Separation Manifold (Fig. 2G), which uses an array of 24 tips to remove chemicals from only the upper interface of the microplate. The custom microplate itself (Fig. 2H) holds the embryos in six corresponding Transplates (yellow) which are capped with a Seplate attachment. The embryos lay between the Transplate and Seplate attachments, which is important for isotonic shocking during fixations (Also illustrated in Fig. 3 as magenta embryos). The microplate sits on a heating/shaking device, allowing for programmable temperatures and shaking speeds.

### The fluid path for *Flyspresso*

A detailed fluid path for *Flyspresso* is illustrated in Fig. 3. The System Piston is a positive-displacement syringe controlled by a solenoid valve, which switches between a vacuum and an N_2_ gas supply to push or pull the blue, internal ethanol-based System Liquid (Fig. 3A). The System Liquid can travel to either the six Chemical Syringes Pumps (Fig. 3B) or the Priming Syringe Pump (Fig. 3C) to control their movement.

Fluidic circuits to the Chemical Syringe Pumps (Fig. 3B) can be opened or closed via solenoid valves, allowing *Flyspresso* to control individual syringes if necessary. The System Liquid is displaced above the Chemical Syringe Pumps, moving them up or down. Plunger displacement pulls the chosen chemical from the Chemical Manifold into the syringe’s chamber and pushes the chemical into the corresponding microplate well below. The directional flow of fluids is passively controlled via passive-check valves.

When a new chemical is pulled from the Chemical Manifold, residual chemicals need to be cleared from inside *Flyspresso.* To do this, the Priming Syringe Pump (Fig. 3C) pushes and pulls the new chemical through the tubing and sends it to Chemical Waste, “priming” the system. A solenoid valve controls whether the path to the Priming Syringe Pump is opened or closed.

A Chemical Waste container (Fig. 3D) is attached to a vacuum, which is also controlled by a solenoid valve. When opened, waste from tubing within *Flyspresso* and from the wells can be aspirated to the container. Passive check-valves prevent the waste from flowing back into the wells or into the syringes.

The Chemical Manifold (Fig. 3E) is a modular and expandable set of reagent bottles attached to solenoid valves. When the desired chemical is selected, the valve is opened, switching from a closed vacuum to ambient air, allowing the chemical to be drawn into either the Priming Syringe Pump or the Chemical Syringe Pumps when the vacuum is released.

### Isotonic shocking

The technological breakthrough with *Flyspresso* is the ability to remove the vitelline membrane—a structure surrounding the outer surface of the plasma membrane of the embryo—in a process known as isotonic shocking^14^. To isotonically shock the embryos, the microplate holds the embryos in Transplate mesh baskets (Fig. 3F). Nested inside the Transplates sit corresponding Seplate Attachments with small inverted openings in the middle. The vitelline membrane is removed by adding methanol from the base of the microplate using the Dispense Manifold and rapidly shaking the microplate. If successful, the vitelline membrane detaches from the embryo, causing the fixed embryos to sink to the base of the microplate, while the membranes float and pass through the cone of the Seplate to the upper surface. The Separation Manifold aspirates fluid from the upper surface and the Dispense Manifold continues to add methanol, creating a one-way upward flow. This one-way flow prevents the debris from recirculating with the successfully shocked embryos—and is a critical step that normally requires extensive manual work. *Flyspresso* is currently the only liquid-handling robot designed to do such steps automatically.

The pipette tips attached to the Chemical Syringe Pumps can add or remove chemicals by displacing the chemical at the base of the wells in the microplate (Fig. 3G, left). These chemicals can additionally be aspirated through the same tip to the Aspiration Manifold (see Fig. 2E) and then to waste. During isotonic shocking (Fig. 3G, middle and right), membranes and embryos that fail to separate float at the methanol phase through the small inverted openings in the Seplate Attachments. The 24 tips from the Separation Manifold (see Fig. 2G) can now remove the upper layer in the Seplate without disturbing fixed embryos at the base of the Transplate.

### The *Flyspresso* pipeline compared to traditional methods

**Figure 4:**
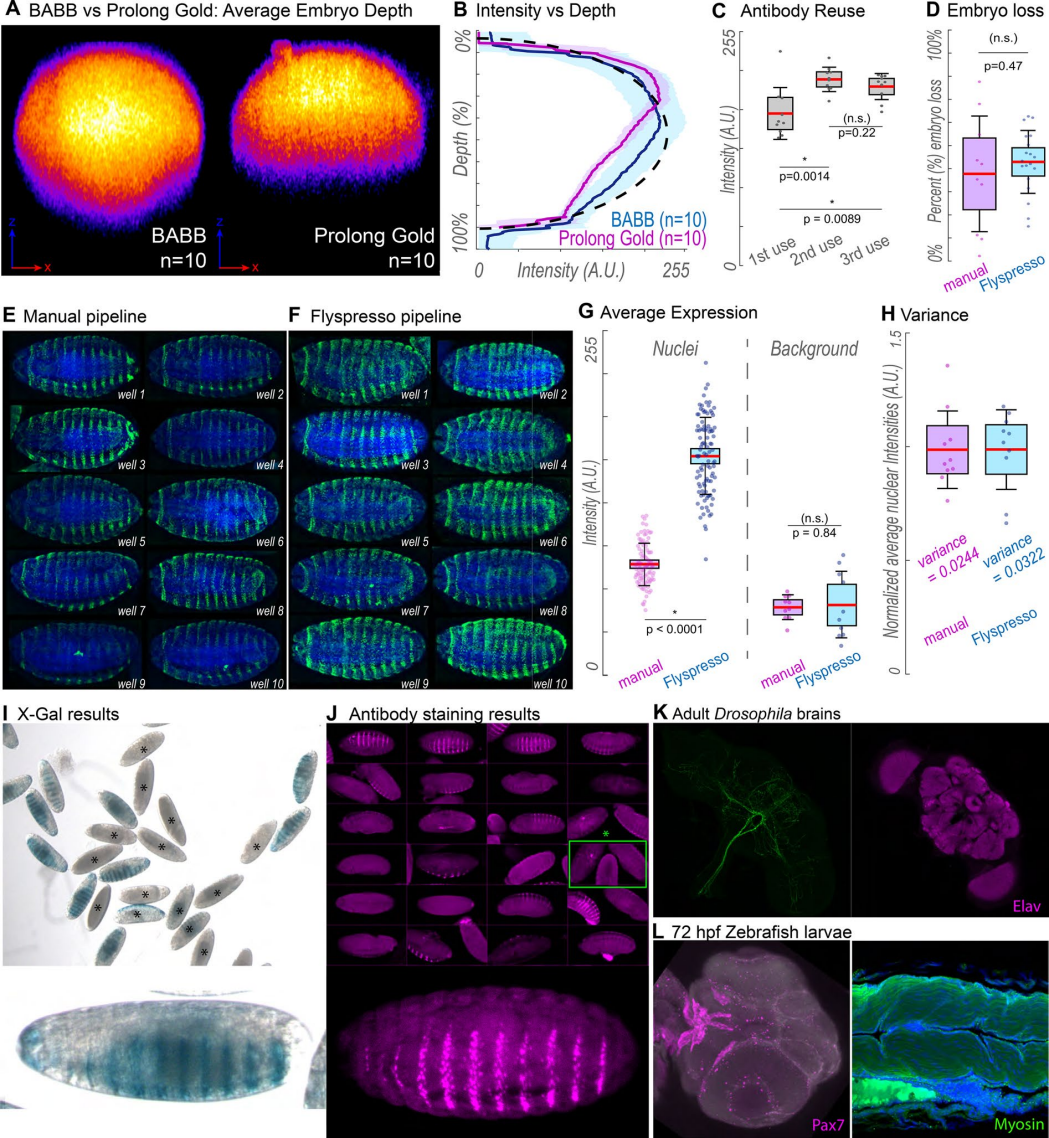
Expected results. (**A**) Resliced z-stacks along the x–z axis. 10 Embryos were composited together for each image. Embryos were either mounted in BABB (left) or Prolong Gold (right). (**B**) Signal intensity going through the mounted embryos—BABB (blue) and Prolong Gold (magenta). Solid colored line indicates the mean, lightly shaded regions are +/− 1 standard deviation. The black dotted line represents the hypothetical shape of an embryo without fluorescent decay. (**C**) Box plots comparing fluorescence intensity after reusing antibody solutions. Each point represents the average expression of an individual embryo (N = 10 embryos per condition). (**D**) Box plots comparing the amount of embryos lost during fixation using manual conditions (magenta, N = 10) or *Flyspresso* (blue, N = 19). Each point represents the percent loss from a single fixation. (**E**) Single representative embryos from 10 embryo pools using manual conditions and (**F**) the *Flyspresso* pipeline. (**G**) Box plots for the average nuclear intensity (left) and background intensities (right). Left: each point represents the average nuclear expression for a single embryo (N = 10 pools, n = 10 embryos per pool, 100 points total) between the manual pipeline (magenta) and the *Flyspresso* pipeline (blue). Right: the average intensity between the stripes for each represented embryo shown in panels e and f for the manual pipeline (magenta, N = 10) and the *Flyspresso* pipeline (blue, N = 10). (**H**) Boxplots for the normalized nuclear intensities, where each point is the average nuclear intensity for each pool for manual methods (magenta, N = 10 pools) and the *Flyspresso* pipeline (blue, N = 10 pools). (**I** and **J**) Reporter construct variant *173-2* for the *E3N* enhancer. (**I**) X-gal staining overview image for *173-2.* Black asterisks (*) indicate embryos not at developmental stage 16 or not in the ventral orientation. (**J**) Antibody staining with *Flyspresso* overview image for *173-2.* Green box indicates embryo clustering. (**K**) Adult *Drosophila* brain co-stained with a neuronal reporter and Elav. (**l**) 72hpf Zebrafish embryo stained with *Flyspresso* for Pax7 (left, magenta) and Myosin (right, green). For all box plots, the red line is the mean, light gray box is the Standard Error Mean (95% Confidence interval) and the whiskers +/− 1 standard deviation. *p* values are for a Student two-tailed t-test.

In Fig. 4, we demonstrate the *Flyspresso* pipeline and how it compares with traditional methods. We first chose to mount embryos in BABB instead of a typical mounting media such as Prolong Gold (Thermo Fisher). We processed two embryo pools in parallel, fixing and staining them for the *Drosophila* Crumbs protein using the monoclonal Developmental Studies Hybridoma Bank (DSHB) antibody: Cq4^15^. One pool was cleared and mounted in BABB, the other mounted in Prolong Gold. We imaged both pools using the same confocal settings. We projected a cross-sectional average of 10 embryos for both conditions (Fig. 4A) and plotted their fluorescence against the embryo’s depth (Fig. 4B) (See methods). Both the cross-section and plot reveal that imaging embryos in BABB allows us to carry out confocal imaging completely through embryos with only a minor decay in fluorescence compared to Prolong Gold.

Like in traditional Drosophila immunostaining^16^, we next demonstrate that expensive primary and secondary antibody solutions can be reused with *Flyspresso*. We used the same primary and secondary antibody solutions three times, and measured the embryo intensities for Crumbs (Fig. 4c). Interestingly, we find that the average fluorescence slightly improves between the first and second stains (*p* = 0.0014), and between the first and third stains (*p* = 0.0089). There was not a significant difference between the second and third staining (*p* = 0.22). The fluorescence likely increases after the first round of staining by acting as an additional blocking step to remove non-specific interactions. This makes reusing antibodies not only cost-effective, but slightly improved the imaging quality for us.

We next compare the amount of embryos lost during manual fixation and with *Flyspresso* by counting the number of embryos before and after fixation (Fig. 4D) (See methods). We manually fixed 10 pools of embryos and 19 pools with *Flyspresso* and found no significant difference (*p* = 0.47) between the percent loss of embryos using manual fixations (38%) and using *Flyspresso* (43%).

We next compare the expression patterns using manual techniques and the *Flyspresso* pipeline. Ten pools of embryos were manually fixed and stained for the *shavenbaby* gene, and ten respectively with *Flyspresso.* Representative embryos of the same age and orientation are shown in Fig. 4E,F. The samples were processed in series, and we found a higher level of fluorescence from embryos stained with *Flyspresso* compared with manual techniques (*p* < 0.0001), but an insignificant difference (*p* = 0.84) in background signal (Fig. 4G). This could be because a larger volume of antibody solution was used, which would allow for more stochastic interactions during the 2-h incubations. When the intensities are normalized by their respective means (Fig. 4H), the variances are very similar (manual variance = 0.0244, *Flyspresso* variance = 0.0322), overall suggesting staining with *Flyspresso* may lead to brighter signal, but there is not a significant difference between the variances from manual preparation or *Flyspresso.*

### Representative expression patterns and problems

We show expected results from both x-gal (Fig. 4I) and antibody staining (Fig. 4J). We stained the same fly line, a variant of the *shavenbaby E3N* enhancer driving the *lacZ* gene. We allowed the flies to lay eggs overnight, creating a range of embryos at different stages on the microplate wells and microscope slide.

While both protocols result in a similar output, the x-gal staining pattern is a much less refined compared to the antibody staining (Fig. 4A,B). The striped pattern is less clear, and the entire embryo begins to take on a dark shade of blue. If the staining solution incubates for too long, eventually the embryos will stain completely blue. The antibody-staining in comparison, shows individual nuclear resolution and a much sharper pattern.

One potential problem with the adaptive feedback microscopy is sample overcrowding within the wells. While the plugin does account for embryos in close proximity and can segment individual embryos using a watershed algorithm, sometimes the microscope cannot correctly decide which embryo to image—in particular if the embryos are overlapping. This is illustrated in Fig. 4J, where the green box highlights a cluster of embryos that were not properly segmented. To circumvent this problem, we recommend no more than 100 embryos per well or using silicone isolators with a larger diameter.

To increase the speed, Job 3 (Embryo focus) is acquired at a very low imaging resolution (128 x 128). The embryo is immediately thresholded and masked, so the details of the stain are not critical. However, if the intensity is too low, the algorithm may have trouble properly rotating and centering directly on the embryo. If your chosen stain is not very intense, a quick solution to this problem is to acquire the final job as a square instead of an elongated rectangle without applying automated rotation to the field of view. Selecting a slightly lower zoom factor (e.g. 0.8) will be needed to fit the entire embryo to the image. The only drawback of rectangular high zoom acquisitions is additional imaging time needed to scan a larger background area. In the future, we envision updating this protocol to implement more complex segmentation algorithms using user-friendly machine learning algorithms^17^.

*Flyspresso* protocols are highly customizable, allowing the implementation of not only *Drosophila* embryo protocols, but protocols for other tissues and model systems. As proof of principle, we adapted the original protocols and carried out antibody staining protocols for *Drosophila* adult brains and *Danio rerio* (zebrafish). We dissected adult *Drosophila* brains using a previous protocol^18^. The brains were stained on *Flyspresso* using the antibody staining protocol described in this work and imaged manually (Fig. 4K). We additionally fixed 72hpf zebrafish larvae manually and antibody-stained them for Pax7 and Myosin using *Flyspresso* (Fig. 4L). The Zebrafish tails were dissected, mounted in 1.5% low melt agarose and covered in PBS.

## Discussion

### *Flyspresso* compared to other commercial robots

Several commercial automated liquid-handling robots exist. Bulk Liquid Dispensers use peristaltic, or syringe pumps to dispense liquids into microplates, require “priming” chemicals to clean the system and require more reagents. Transfer devices use pipettes to transfer liquids to microplates but require time-intensive cleaning phases or consumable pipette tips. Both Bulk Liquid Dispensers and Transfer Devices are components of “Liquid Handling Stations,” which contain many individual dispensers for a specific protocol^8,19^. Conversely, Microplate Washers like *Flyspresso* are independent Liquid Handling Stations similar to Bulk Liquid Dispensers, but also remove liquids using an Aspiration Manifold^19^. The majority of Microplate Washers are designed for cell-work and not optimized for the unique demands of whole organismal studies.

We compare *Flyspresso* to other liquid-handling robots that can stain *Drosophila* embryos. The company Intavus (https://intavis.com/) sells two robots designed for whole-embryo immunohistochemistry: the Insitupro VSi and the Biolane HTI 16Vx.

The Insitupro VSi is a Transfer Device dependent on a peristaltic pump-controlled pipette. Both the Insitupro VSi and *Flyspresso* are capable of heating and shaking microplates. The InsituPro VSi has advantages to *Flyspresso* since it can also work on microscope slides and hold 60 samples compared to the 24 that *Flyspresso* holds, which we optimized for holding enough embryos per well. *Flyspresso*, however, is a much smaller device compared to the InsituPro VSi, and occupies a relatively small space in a chemical fume hood or benchtop. Furthermore, the Chemical Manifold on *Flyspresso* is easily expandable, allowing *Flyspresso* to hold more than 18 chemical reagents, the current limit for the Insitupro VSi.

The Biolane HTI 16Vx is a Microplate Washer, like *Flyspresso*, but uses a peristaltic pump instead of a syringe to transfer chemicals. Equipped with 16 different buffer positions, a microscope slide washer, and the ability to wash up to 384 samples simultaneously, the Biolane HTI 16Vx is optimized for high-throughput techniques such as in-situ hybridizations*.* The form factor, however, of a dense 384-well microplate is not conducive to whole organism studies, as not enough embryos would fit in each well and transferring embryos to 384 wells is simply not practical due to time constraints and the high risk of sample cross-contamination.

With our current setup, embryos can be collected in our custom-designed collection chambers (_24W Fly Deck .iam), where eggs are laid directly in the Transplate attachments, which easily detach to be loaded directly into *Flyspresso.* Using the collection chambers and Transplates for *Flyspresso,* samples would never have to be transferred. Another difference between the products, however, is that the Biolane HTI 16Vx cannot carry out embryo fixations. This is because liquid runs through the Transplate meshes during washes and cannot be removed from specific interphases, making isotonic shocking impossible.

Finally, given the small footprint of a *Flyspresso*, it is possible to put the entire device into a standard incubator. For cell culture-based experiments, this allows the integration of tissue culture, fixation, and staining. The small footprint also allows the ability to array multiple units, making the system modular and scalable.

### Expertise needed to build and operate the pipeline

To build *Flyspresso*, an engineer or someone equivalently trained, is needed who can read Computer-Aided Design (CAD) files and component schematics. The skilled individual must be able to assemble the individual components. Additionally, somebody with an understanding in programmable microcontrollers such as Arduino or Raspberry Pi, who can additionally read and build circuit schematics, is necessary. Operating the robot and the protocol can be performed by any scientist that can complete the protocol manually.

The pipeline for feedback microscopy is composed of several tools responsible for managing the multistep workflow, image analysis routines and communication to the microscope. The guidelines for how to construct similar pipelines and use them on different microscopes are provided in the documentation for the core AutoMicTools library (https://git.embl.de/halavaty/AutoMicTools). The project-specific part of the pipeline is implemented as a Fiji plugin. The source code for the plugin is provided and can be adopted to particular needs by the person having experience for writing either Java plugins or scripts in Fiji.

### The current limitations of our pipeline

We encourage readers to build upon the current model for *Flyspresso*, as there are limitations to *Flyspresso*’s design. First, Bulk Dispensers and Microplate Washers require a “priming” step, where a new liquid must clear the tubing before added to the samples. The waste from this process could be reduced if a peristaltic pump is used, since they can rotate in both directions to recover lost chemicals^19^. A special reagent port could be added directly to the dispense manifold that eliminates any tubing from the Chemical Manifold. A separate dispense circuit for high value reagents (antibodies) can be added which eliminates the need for priming. *Flyspresso*, however, is currently designed to circumvent expensive reagent losses by a pause step to add antibodies to the microplates manually.

Second, *Flyspresso* can hold 24 samples, which share six different chemical wells. Since the embryos still share six wells, only six different chemical conditions can be tested (i.e. antibodies, chemical concentrations, etc.). Modifications to the *Flyspresso* design could be made to optimize the liquid-handler for different experimental procedures.

Third, the initial washing and bleaching of embryos is still done manually, since bleach is corrosive to the current materials. In the future, fabricating *Flyspresso* out of different materials would make this possible.

Fourth, liquid levels in the reagent bottles are manually monitored. To ensure that *Flyspresso* does not exhaust all reagents or to identify potential leaks in the tubing, sensors could easily by attached to the bottles to communicate directly with the Arduino microcontroller.

Another place to improve is the mounting of samples for microscopy. Currently, the samples must be removed from each well manually for imaging. In the future, it should be possible to integrate the robotics directly with microscopy using fluidic based approaches, collaborative robots, or imaging embryos directly in the microplate ^20,21^.

## Conclusion

We have demonstrated *Flyspresso* to be an alternative to other conventional liquid handling robots. We can carry out *Drosophila* embryo fixations for antibody staining, and show that samples stained with the robot can automatically be imaged using our adaptive feedback microscopy pipeline. We hope that others will reproduce and build upon *Flyspresso* and the automated pipeline, not only within the *Drosophila* community but as a community of Biologists as a whole.

## Methods

### Software

Computer-Aided Design (CAD) files can be viewed using any standard program such as Solidworks or Autodesk Inventor. A free CAD file viewer, eDrawings is available here:

https://www.edrawingsviewer.com/

Autodesk also has a free viewer:

https://viewer.autodesk.com/

Circuit board schematics can be viewed using the free open-source program KiCAD, which can be downloaded here:

https://kicad-pcb.org/download/

The software: Eagle, is also free and can be used to view the schematics:

https://www.autodesk.ca/en/products/eagle/overview

Installation and usage instructions for the adaptive feedback microscopy pipeline can be found here:

https://git.embl.de/grp-almf/feedback-fly-embryo-crocker

The documentation page in this repository contains the detailed protocol as well as the installation guidelines for the protocol and its dependencies: the core AutoMicTools library (https://git.embl.de/halavaty/AutoMicTools) for Fiji and the MyPic macro^13^ used as a communication interface between the pipeline and ZEN Black software controlling the microscope.

Operating software for Flyspresso can be downloaded here:

https://github.com/janelia-pypi/hybridizer_python/tree/digital

### Solution setup

- Antibody Fixative—4.6% Paraformaldehyde (PFA) (Electron Microscopy Sciences 15710) and 25 μM EGTA (Sigma-Aldrich E6758-100g) in PBS. The PFA should be added fresh before every fixation.
- BABB—1 part benzyl alcohol (Sigma-Aldrich 305197-1L) and 2 parts benzyl benzoate (Sigma-Aldrich B6630-1L).
- Blocking Solution—1:5 Western Blocking Reagent Western Blocking Reagent (Roche SKU 11921673001) and PBT solution.
- Fly Saline—0.1 M NaCl (Sigma-Aldrich 1064041000) and 0.04% Triton X-100 (Sigma-Aldrich X100-100ML) in sterile water. Fly Saline can be prepared ahead of time and stored at room temperature.
- PBT—PBS and 0.1% Triton X-100. PBT can be prepared ahead of time and stored at room temperature.
- X-gal Fixative—2% Formaldehyde (Sigma F8775-25ML) and 0.2% Glutaraldehyde (Sigma G5882-50ML) in PBS. X-gal Fixative can be prepared ahead of time as a 10X solution and stored at − 20 °C until needed.
- X-gal Staining Solution—5-bromo-4-chloro-3-indolyl-ß-D-galactosidase (Invitrogen B1690-1G), 20 mg/mL DMF (Sigma D4551-250ML), 400 mM potassium ferricyanide (III) (Sigma-Aldrich 244023-100G), 400 mM potassium ferrocyanide (Sigma-Aldrich P3289), 200 mM magnesium chloride (Merck 1058331000) in sterile water. Staining solution can be stored at − 20 °C until needed.

### Egg collection and chorion membrane removal (30 min)

Prepare apple-juice agar (Sigma-Aldrich A1296) and a yeast paste mixture. Load flies into the collection chambers with the Transplates. Place the loaded egg collection chambers at 25 °C and let the flies lay overnight. Remove the Transplates from the fly chambers. In the Transplate, wash embryos with fly saline. Remove any dead flies from the Transplates using forceps. Place the Transplates in a 50% bleach solution (Sodium Hypochlorite, Sigma-Aldrich 1056142500) in a shallow petri dish for 90 s. Use a Pasteur pipet to mix the bleach solution. Rinse embryos with water. Load the Transplates embryos into the custom microplate, and add the Seplate attachments on top.

### X-gal fixation and staining (3 h)

*Flyspresso* adds 2 mL of X-gal Fixative and 2 mL of Heptane (Sigma-Aldrich 246654-1L) to each well (6 wells, 24 mL total). The embryos fix for 20 min, shaking at 200 rpm. Fixing samples for too long can denature β-Gal. We optimized these X-gal staining conditions for a specific gene expression pattern. Consider shortening or lengthening the fixation period to adjust the signal and noise of expression.

During the pause step, remove the Transplates and blot the screens of the Transplates with a paper towel. This ensures that all heptane is removed. Wash the samples three times in PBT, shaking at 200 rpm, for 10 min each. Add the X-gal Staining Solution (6 wells, 24 mL total) and incubate for 2 h at 37 °C. Wash the samples three times in PBT, shaking at 200 rpm, for 10 min each. Use scissors to remove the end of the pipette tip to obtain a more accurate amount of Triton X-100. Image the embryos directly from the plate using a Leica DFC420C Digital Camera.

### Antibody staining (7 h)

*Flyspresso* adds 4 mL of Antibody Fixative, and 4 mL of Heptane to each well (6 wells, 48 mL total). The embryos fix for 25 min, shaking at 250 rpm. Fixing samples for too long can denature proteins you wish to stain. To improve the antibody staining conditions, consider shortening or lengthening the fixation period. Blot the bottom screens of the Transplates with a paper towel during the pause step. This ensures that all heptane is removed and will increase the efficiency of the isotonic shocking.

Add Methanol (Merck 1060091000) to the wells (isotonic shocking). Aspirate from the upper interface to remove membranes and embryos that failed to separate, and wash at least 3× in Methanol. The more Methanol washes, the better the staining and the longer samples will hold in storage. Samples can be stored at – 20 °C for at least one year.

Serially rehydrate the embryos in PBT. Add the blocking solution (6 wells, 24 mL total). Block for 25 min. During the pause step, add the primary antibody diluted in blocking solution, 4 mL per well (6 wells, 24 mL total). Antibodies and their dilutions used in this study are as follows: Beta-Galactosidase (1:500, abcam ab9361), RFP (1:500, MBL PM005), Crumbs (1:10, DSHB Cq4 Supernatant), ELAV (1:20, DSHB Elav-9F8A9), Pax7 (1:4 DSHB PAX7), Myosin (1:4, DSHB F59). We recommend making the antibody mixture as a master mix of at least 25 mL of antibody solution. While it is not necessary, moving the Transplates to a new microplate at this step will improve staining accuracy, as residual PBT can persist in each well. The microplate can also be switched again before adding the secondary antibody.

For staining the Zebrafish larvae and *Drosophila* brains in Fig. 4, the microplate was placed at 4 °C and incubated for 16 h (overnight). When troubleshooting antibodies, overnight incubations can also increase the signal-to-noise ratio.

Samples were washed twice in PBT. Add the blocking solution (6 wells, 24 mL total) and block for 25 min. During the pause step, add the secondary antibody diluted in blocking solution, 4 mL per well (6 wells, 24 mL total): AlexaFluor 488 and 633 (1:500, Invitrogen). Make the antibody mixture as a master mix. We recommend making at least 25 mL of antibody solution. Move the Transplates to a new microplate for more accurate staining. Samples should be kept at this point in the dark by using the *Flyspresso* cover. Samples are afterwards washed in PBT.

### BABB clearing and mounting antibody-stained samples (48 h)

If mounting samples in water or glycerol-based media, mount embryos as normal. If using BABB, samples are serially dehydrated into Ethanol (Merck 1009831000). Manually transfer embryos to 1.5 mL Eppendorf tubes. Wash the samples twice in BABB for 10 min each. The embryos will become fully transparent in the BABB. When washing, allow embryos to sink for at least 30 s. Aspirate to the 100-µL mark on the test tube to avoid aspirating samples. Let the samples incubate overnight in BABB.

Divide a silicone isolator and apply with an additional isolator to a microscope slide (Fig. 1I). Add 100 µL of BABB/Embryos to each well. Allow embryos to sink. Connect the wells with BABB and cover with a coverslip. This may take some practice. The slower the better. Lay the coverslip from left to right. Avoid bubbles. The closer the coverslip size matches the silicone isolator surface the better. If you cannot find a coverslip close to the size, we recommend using a glass knife. Immediately seal the perimeter with a coat of clear nail polish (Maybelline Express Manicure—10ML). Lay the slide in a fume hood to allow the nail polish to set for 10 min. Add two more coats of nail polish (apply generously) around the perimeter. Let the slide sit for at least 24 h for embryos to settle. Samples can be stored in the dark at room temperature for a week. The longer the wait, the greater the risk of the fluorophores losing fluorescence and air leaking into the wells, which will ruin the samples.

### Adaptive feedback confocal microscopy (timing variable)

Access to all codes, installation, jobs, and operating the adaptive feedback microscope can be found using the following link:

https://git.embl.de/grp-almf/feedback-fly-embryo-crocker.

After the nail polish has dried, load the microscope slide onto the confocal microscope. The following steps describe a quick start of the pipeline (for the complete manual refer to the documentation in the repository):

1. Launch the microscope and ZEN Black software.
2. In ZEN Black, create the four job files based on the image metadata from the template jobs we provide (DE1: autofocus, DE2: low zoom tile scan, TR1: embryo focus, and TR2: result). Adjust your imaging parameters such as laser intensity, gain channels, etc. appropriately for your protocol. **Note:** please keep in mind that with ZEN Black systems, it is not a good idea to reuse jobs from the files acquired on a different microscope (even if it is the same model), as it often leads to the microscope crashing or malfunctioning. This is why we recommend using our metadata only for creating your own job files and not directly using our template files.
3. Start the MyPic macro in ZEN Black,
  i. In the top menu bar, select the “Macro” category and click on “Macro…” item.
  ii. In the “Edit Macro” panel, click the “Load” button and select the file: MyPiC.lvb.
  iii. When loaded, press “Run” in the same dialog, The MyPic window will appear on the screen.
4. Click on the “JobSetter” located in the top left corner.
5. In the JobSetter window, click on the folder icon and select the four pre-saved lsm or czi files to load the metadata for all imaging jobs.
6. To view the imaging settings of a job in the ZEN GUI, simply select the job of interest and click the button “ZEN” in the JobSetter window. You can change the parameters of the job in ZEN. To update the settings, make the changes in ZEN and in the MyPic window, click the “Macro” button in the JobSetter window.
7. Specify the order of the imaging jobs in MyPic.
  i. Select the Autofocus (Job 1) and Low zoom overview (Job 2) jobs as the first and second default jobs (“Default” tab).
  ii. Select the Embryo focus job (Job 3) in the “Trigger1” tab
  iii. Select the Result job (Job 4) in the “Trigger2” tab
8. Specify the default imaging positions in the “Default Positions” tab. For our sample design, we use the following options:
  i. Select the “Grid” option at the top
  ii. Specify well format (e.g. 4 × 3) and distance between wells
9. Mark the reference position
  i. Navigate the stage to the top left well of the slide and make sure that it is in focus for the first imaging job (Autofocus).
  ii. Mark this position in the “Default Positions” list by clicking the “Mark” button.
10. Configure automated data saving in MyPic (“Saving” tab)
  i. In the “Root Directory” settings, add/create a directory to store the acquired images, subdirectories, and imaging settings.
  ii. In the “Base File Name” window, enter a unique ID name for the run. It is essential to change either the “Root Directory” or the “Base File Name” each time you run the automation. If you do not, the imaging data of previous experiments will be overwritten and the coordinates of the embryo positions will not be identified correctly.
11. Launch Fiji/ImageJ with pre-installed plugins and libraries.
12. Embryos can be automatically located by the pipeline, or the user can select which specific embryos to image at the beginning of the protocol. **Note**: because the overview scan is acquired at 5X resolution, there is still a fair amount of background illumination in the embryos, regardless if they are stained. The automated selection algorithm is based on an automated thresholding procedure, which is able to mask all embryos in the foreground from the background. If embryos are needed at only a particular stage, orientation, or the algorithm is biasing towards selecting embryos with higher intensity for whatever reason, we recommend using the manual embryo selection tool.
13. In Fiji, carry out the following depending on if you want to run automated imaging (i) or manually select the embryos (ii):
  i. Automated embryo selection
    1. **Select Plugins Auto Mic Tools Projects Fly Embryo Screen Automated pipeline** to allow the pipeline to identify the embryos automatically.
  ii. Manual embryo selection
    1. **Select Plugins Auto Mic Tools Projects Fly Embryo Screen Semi-automated pipeline (manual embryo selection)** to select the embryos yourself.
14. Select the root directory where the experimental results are stored (this must be the same directory chosen in MyPic).
15. Multiple dialog boxes will appear to specify pipeline parameters. The user may have to change these settings depending on how the samples are mounted on the slide. The given numbers are the pre-settings used during the screen from Fuqua et al., 2020^10^. Do not press “Stop Monitor” until complete with imaging.
16. Return to the MyPic macro and click the start arrow. If automated embryo selection was chosen, the protocol will now run completely autonomously. If manual selection was chosen, after the second job (Low zoom overview), is run, a dialog box will appear and an image will open in a Fiji window. Select individual embryos with the left-click on the mouse, followed by pressing “t” on the keyboard to add the positions to the Fiji ROI Manager. When all the embryos are selected, press the “OK” button on the Select Embryo Positions dialog box. Repeat these steps for every well. After embryo positions are manually selected for every well, the microscope will begin the automated feedback imaging. Depending on imaging parameters, this can last more than 72 h.

### BABB vs Prolong Gold analysis

Two pools of embryos were stained with the Crumbs antibody (1:10, DSHB Cq4 supernatant) and an Alexa Fluor 488 (1:500, Invitrogen) in the microplate. One pool was mounted on the multiwell slide in Prolong Gold and the other eluted into ethanol, cleared in BABB, and mounted on the multiwell slide. Ten embryos were imaged for each condition.

In Fiji, stacks were resliced using the **Reslice[/]** command to view the embryos along the x–z axis. Resliced images were projected using the **Z Project** command for the Max Intensity.

To create the composite representations, each projection was concatenated together using the **Concatenate** command and projected again with **Z Project** for the Average Intensity. Brightness and contrast were enhanced for clarity. Because BABB and Prolong Gold have different indexes of refraction^22^, composite images were stretched based on the following formulas previously derived^23,24^:

$$\frac{d{^{\prime}}}{d}= \frac{\mathrm{tan}\left({\mathrm{sin}}^{-1}\frac{0.5 NA}{{n}_{1}}\right)}{\mathrm{tan}\left({\mathrm{sin}}^{-1}\frac{0.5 NA}{{n}_{2}}\right)}$$

To create the plot in Fig. 4B, the concatenated stacks were rotated − 90° using the **Rotate** command. A box was drawn through the center of the embryo using the “Rectangle” selection tool. Intensities within this region of interest were measured using the **Plot Profile** function. Data were normalized in both intensity and embryo length and plotted using MATLAB.

### Antibody reuse analysis

Embryos were stained with the Crumbs antibody (1:10, DSHB Cq4 supernatant) and an Alexa Fluor 488 (1:500, Invitrogen). Antibody solutions were placed at 4 °C overnight, reused the following day, and used three times in total. Ten embryos were imaged for each condition. In Fiji, images were projected from the beginning to halfway through the embryo using the **Z Project** command for the Max Intensity. The embryo was selected using the **Threshold** command and the average intensity was measured. Two-tailed t-tests were used to compare the data. Boxplots were plotted in MATLAB using the NotBoxPlot tool^25^.

### Embryo loss analysis

Photos of the embryos in meshes were taken after being collected and bleached. After fixing using manual techniques or *Flyspresso,* another photo was taken of the embryos. For pre-fixed embryos, we used Fiji to mask regions that contained embryos using the **Threshold** command and measured the total area covered by embryos. To estimate the number of embryos, the sizes of ten embryos clearly defined by the thresholding were measured and the total area was divided by the average size of these ten embryos. For post-fixed embryos, a mask was made using the **Threshold** command. Boundaries were refined using the **Watershed** command. Embryos were counted using the **Analyze Particles** command and particles within a range of 3–300 squared-pixels were counted. The average losses were calculated and plotted in MATLAB using the NotBoxPlot tool^25^. The datasets were compared using a two-tailed t-test.

### Well variance analysis

Ten pools of embryos were fixed and stained manually and ten pools of embryos were fixed and stained using *Flyspresso.* Samples were stained for dsRed expression, driven by a BAC for the *shavenbaby* (*svb*) locus using the RFP primary antibody (1:500, MBL PM005) and an Alexa Fluor 488 (1:500, Invitrogen). From each pool, ten images for embryos around stage 16 were manually selected and imaged using the adaptive feedback confocal pipeline (100 embryos per condition). Nuclear intensities were measured in Fiji by creating a mask using the **Threshold** command. Boundaries were refined using the **Watershed** command. Nuclei were selected using the **Analyze Particles** command, particles within a range of 5–50 squared-pixels were chosen, and the average intensity within each nucleus was measured.

To measure the background, the **Make Inverse** command was used to create an inverse selection. The DAPI channel was thresholded so the new selection was only measuring non-unclear intensities within the embryo.

To measure the variance, the average nuclear intensities were each divided by their respective average and the sample variance was calculated. All data were plotted in MATLAB using the NotBoxPlot tool^25^. The datasets were compared using a two-tailed t-test.

### Animal models

This study was carried out in compliance with the ARRIVE guidelines.

For the *Drosophila* embryos, adult *Drosophila melanogaster* carrying the *attp2* insertion were transformed with reporter constructs and outcrossed into *w*^*1118*^ lines as previously described^10^. Adult *w*^*1118*^ flies were used for brain dissections.

Wild type AB Zebrafish were kept at 27–28 °C in a 14/10-h dark cycle. We used zebrafish embryos after 72 hpf for fixation and antibody staining. To prevent pigmentation, 0.003% 1-phenyl-2-thiorea was added at 24 hpf. The required project approval has been obtained from the EMBL IACUC Committee in accordance with the requirements of the German national authorities regarding experiments with vertebrate animals.

## Data Availability

We provide all .ipt, .stp, .dwg, and .iam files, which contain the robot components. The final assembly of *Flyspresso* is the CAD file: *_24W.iam*. CAD files can be found here, along with the actual protocols *Flyspresso* uses for x-gal and antibody staining: https://github.com/tfuqua95/Flyspresso-CAD-files Software for operating *Flyspresso* can be located here: https://github.com/janelia-pypi/hybridizer_python/tree/digital PCB schematics for all circuits and the Arduino microcontroller can be found here: https://github.com/janelia-modular-devices/mixed_signal_controller The automated feedback microscopy pipeline, installation guide, and an operation manual can be found under the following link: https://git.embl.de/grp-almf/feedback-fly-embryo-crocker The original images used for experiments and data files for their analysis are available for download and are indexed here: https://www.embl.de/download/crocker/flyspresso/index.html
